# Annotations

**Published:** 1891-07-11

**Authors:** 


### Annotations.

**Our Imperial Visitor: A Medical Greeting.**

To the doctor and the physiologist a man helpless and sick in his bed is but a man, be he emperor or be he slave. An emperor "bleeds if we prick him," as Shakespeare reminds us; he must be nourished with carefully-prepared slops when he is sick, like an ordinary man, and his dose of medicine must be the same, both in quantity and quality, as if his name were Robinson, or Jones, or Smith. Nobody knows this better than the emperor himself, and especially if he happen to be of the tough and practical stock of the Hohenzollerns. But, notwithstanding the facts of physiology, the doctor does not think less well of an emperor than other men think of him, if the emperor be worth thinking well of. The greeting which we have to offer to our Imperial visitor is not less sincere and full of admiration than that of many who will think him made of other clay than themselves. The Emperor of Germany has established for himself a special claim upon men of science and men of honesty. He has proved himself first of all a man. He was born to be a king, and he dares to be a king: that is kingly. He was born to rule and govern, and he dares to rule and govern: that is royal. A man, whatever be his position, should have the courage of his position. If he feels himself capable, he should do in actual deeds that which his position demands. The man who does this always commands the sympathy and admiration of the best part of the world. The German Emperor began his Imperial work, as some people thought, impulsively. Such was the appearance of himself and his doings that was presented to the world. He seemed to be rash and somewhat wilful. But he has done what physicians have to do every day; he has had the courage and the honesty to retrace his steps when he has discovered himself to be moving in a wrong direction. This is one of the special characteristics of the young Emperor which commends him to all thinking, honest, and patriotic men. Nothing is so foolish, so wicked, or so unkingly as blind obstinacy. In past times rulers of people required courage and the power of leadership on the battle field. In these times rulers of men require intellectual capacity, the power to see and understand, the steadfastness to move right on, or the bold discretion to turn right back. At the present moment the German Emperor stands well with the very class of people with whom it is the highest possible honour to stand well. The best sort of Englishman loves two things, liberty and order. Licence and anarchy he hates. The German Emperor at present stands for liberty and order as they are understood in Germany. That is what he should do. The scientific medical man cannot but regard with admiration a powerful Emperor who in politics carries out the principles which have proved the truest and safest in medicine: the principles, namely, of doing always the best and the honestest which a careful study of the facts, near and remote, prompts us to do.

**Scientific Lunatics.**

A considerable sensation has recently been created by the announcement that a French doctor had inoculated certain patients with cancer germs without their consent, and that the patients had become cancerous. The leaders of medicine in Paris promptly repudiated their insane professional brother; and it seemed as if the matter would end there. But the Berlin correspondent of the *Daily News* telegraphs that "it has been discovered that similar experiments have taken place in Germany, and particularly in Berlin; and a German doctor charges Professors Von Bergmann and Hahn as being among those who have practised such operations." It was charitably hoped that the French experimenter was the only man in Europe whose brain and conscience were alike so feeble as to permit him to inoculate human beings with cancer products. If the correspondent, whose telegram we have quoted, be correctly informed, it is clear that there are other criminal lunatics in the medical profession as well as the miserable Frenchman. The question at issue admits of but one kind of argument, the argument of the policeman and the prison. There are no physicians and surgeons in the world who are more sincerely anxious for the true and rapid progress of medicine than the medical men of this country; and there are none more competent to conduct experiment, to guide experience, and to reason soundly. But we are absolutely confident that there is no single doctor in Great Britain of any professional status or personal worth who would for a moment think of practising such monstrous and diabolical tricks upon his patients as are reported from France and Germany. One such act would be sufficient to bring irretrievable professional ruin upon its perpetrator. Whatever may be the prominence of certain persons who have taken part in these villainous experiments, the medical profession, both in France and Germany, and indeed in every other country, should not rest content until all the facts are brought to light; and if it can be proved that the things reported have actually been done, tho doers of them should be promptly expelled, not only from the ranks of medicine, but from those of honest men. A prison is much too wholesome a place for such infamous scoundrels.

**A Call to Intelligent Philanthropists.**

The London Hospital has just been compelled to spend £26,000 in additions and improvements. We have seen the additions, and can testify that they are improvements, and that they were absolutely necessary. Those who think of giving money to help to pay for the improvements had better go down to Whitechapel and see them before writing out their cheques. They will probably give twice as much after seeing them as they would have done without passing through that preliminary experience. But in case any benevolent lady or gentleman has not time to go down to Whitechapel, or is willing to take our voucher for the facts, we will say in a word exactly what has been done. The first thing that has been provided for the comfort of the patients and for the general amenity of the hospital is a suitable approach and covered entrance. There is hardly a poor half-clothed man or woman in the whole of East London but will bless the managers of the London Hospital for this new protection against the summer's rain and the winter's cold. The next thing provided has been a receiving-room, with all the new and needful appliances. Let the philanthropist picture to himself the numerous desperate cases of every kind that are constantly being sent to the London Hospital; the accidents on the river and the accidents in the street; the victims of drunken brawls and of domestic cruelty; the bruised and battered women, and the children already more dead than alive from fever, starvation, and dirt. To such the entrance into the new and spacious receiving room will often be like passing from the black midnight of the inferno to the beautiful opening dawn of sunshine and hope. There is also a new operation theatre, a gruesome apartment, but often a place of deliverance from death, and of the beginning of a new life. Lastly, there is a new clinical theatre and college hall, where those teachers whose minds are charged, and almost overcharged, with the tremendous daily experiences of the London Hospital, tell to hundreds of eager students what they have seen with their own eyes and handled with their own hands. In that theatre also the students may see almost every day what they can hardly see with such fulness of detail in any other clinical theatre in the world. The London Hospital was compelled to make these additions and improvements. The necessities of its neighbourhood, which are urgent beyond description every day, and the responsibilities of its unique position as an unrivalled school of clinical medicine, left it no choice. If the managers had hesitated they would have been unworthy of their profoundly responsible position. They have spent the £26,000 required, and they have spent it well. They look to philanthropists as intelligent and as responsible as themselves to give them the money. They cannot afford a penny of it out of their annual income, for every new year adds to the beds of the hospital and increases the number of in-patients, sometimes by many hundreds. In 1890 nearly six hundred more in-patients were received than in 1889; and the total number of in-patients now taken in annually is between nine and ten thousand. In many respects the London Hospital is almost the noblest charity in the world. It is situated exactly where it is wanted. It is managed with such skill, kindness, and economy as have rarely been equalled, and never surpassed. The noble institution is like a brooding providence to the sick poor of the East-end of London; to men and women of all nations, characters, and creeds. For our part we feel that its claims are irresistible. Nothing would give us greater joy than to hear that the whole of the £26,000 needed had been subscribed by the readers of The Hospital within a week of the issue of this appeal.

